# Assessing Progress towards Public Health, Human Rights, and International Development Goals Using Frontier Analysis

**DOI:** 10.1371/journal.pone.0147663

**Published:** 2016-01-26

**Authors:** Jeanne Luh, Ryan Cronk, Jamie Bartram

**Affiliations:** The Water Institute, Department of Environmental Sciences and Engineering, Gillings School of Global Public Health, University of North Carolina at Chapel Hill, Chapel Hill, North Carolina, United States of America; UNAIDS, TRINIDAD AND TOBAGO

## Abstract

Indicators to measure progress towards achieving public health, human rights, and international development targets, such as 100% access to improved drinking water or zero maternal mortality ratio, generally focus on status (i.e., level of attainment or coverage) or trends in status (i.e., rates of change). However, these indicators do not account for different levels of development that countries experience, thus making it difficult to compare progress between countries. We describe a recently developed new use of frontier analysis and apply this method to calculate country performance indices in three areas: maternal mortality ratio, poverty headcount ratio, and primary school completion rate. Frontier analysis is used to identify the maximum achievable rates of change, defined by the historically best-performing countries, as a function of coverage level. Performance indices are calculated by comparing a country’s rate of change against the maximum achievable rate at the *same* coverage level. A country’s performance can be positive or negative, corresponding to progression or regression, respectively. The calculated performance indices allow countries to be compared against each other regardless of whether they have only begun to make progress or whether they have almost achieved the target. This paper is the first to use frontier analysis to determine the maximum achievable rates as a function of coverage level and to calculate performance indices for public health, human rights, and international development indicators. The method can be applied to multiple fields and settings, for example health targets such as cessation in smoking or specific vaccine immunizations, and offers both a new approach to analyze existing data and a new data source for consideration when assessing progress achieved.

## Introduction

Public health, human rights, and international development goals and targets such as the Millennium Development Goals (MDGs) and the recently adopted Sustainable Development Goals (SDGs) are established to improve the welfare of the world’s poorest people by alleviating hunger and poverty, improving health and education, empowering women, promoting equality, and ensuring environmental sustainability. Progress towards the associated targets is monitored through indicators that typically track a country’s *status*, defined as the level of attainment or coverage. For example, one of the indicators used to assess progress towards MDG Target 5b, to “achieve, by 2015, universal access to reproductive health” is “contraceptive prevalence rate” [1]. Using status alone, the 2010 contraceptive prevalence rates for Armenia, Burkina Faso, and Colombia were 54.9%, 16.2%, and 79.1%, respectively [2], suggesting that Colombia is making the greatest progress while Burkina Faso is making the least. However, using status to compare countries does not reflect a country’s performance, as a country can be increasing, decreasing, or stagnating in its coverage level, which we refer to as progression, regression, and stagnation, respectively.

An alternative approach to monitoring progress is to assess trends in status over time, i.e., *rates of change*. Rates of change identify whether a country is moving towards a target and provides information on how fast a country is improving or regressing over time. Using contraceptive prevalence rates from 1990 (the MDG baseline year) to 2010, the rates of change using linear regression for Armenia, Burkina Faso, and Colombia are -0.13, 0.54, and 0.64%/year, respectively. This indicates that Armenia is moving away from providing universal access to reproductive health care (i.e., regression), and despite the low status of Burkina Faso, it has been making progress towards MDG Target 5b. However, status cannot be completely disregarded as it affects a country’s rate of change. This is because countries at very high levels of coverage can only make small improvements as they approach 100% because the remaining unserved become more difficult to reach, and countries at very low levels of coverage make little initial progress as the systems, policies, and infrastructure required are not yet in place.

With the MDGs having ended and the recent adoption of the SDGs and its associated targets, it is essential that a method is available to assess progress and fairly compare the performance of countries with one another. One approach that accounts for different levels of status is the use of frontier analysis to compare the rate of change of a country against the rate of the historically best performing countries at *similar levels of coverage*. One recent study applied this approach to water and sanitation and demonstrated its effect on impacting policy and practice [3]; however, it has seen limited use in other public health and international development fields. In this paper, we briefly review the principles of frontier analysis, the steps needed to perform frontier analysis, and the application of this method to three MDG and SDG targets which are relevant to global and community health: maternal mortality ratio, poverty headcount ratio, and primary school completion rate.

## What Is Frontier Analysis?

Frontier analysis is based on the principles of data envelopment analysis (DEA), which is a non-parametric method used to evaluate and compare the efficiency of decision making units (DMUs) such as hospitals, schools, and banks and to define a “best-practice frontier” or “benchmark” for operations management [4]. In simple terms, Seiford and Thrall [5] refer to the best-practice frontier as a floating piece-wise linear surface that sits *on top* of all data points (i.e., envelops the data) and which consists of the best-performing DMUs. The efficiency (which for our purposes, we refer to as performance) of a DMU is then calculated as the ratio of its distance from the best-practice frontier. The assumption is that if a DMU with input *x* can produce output *y*, then other DMUs with the same input *x* should also be able to produce the same output if they were operating efficiently.

**Fig 1:**
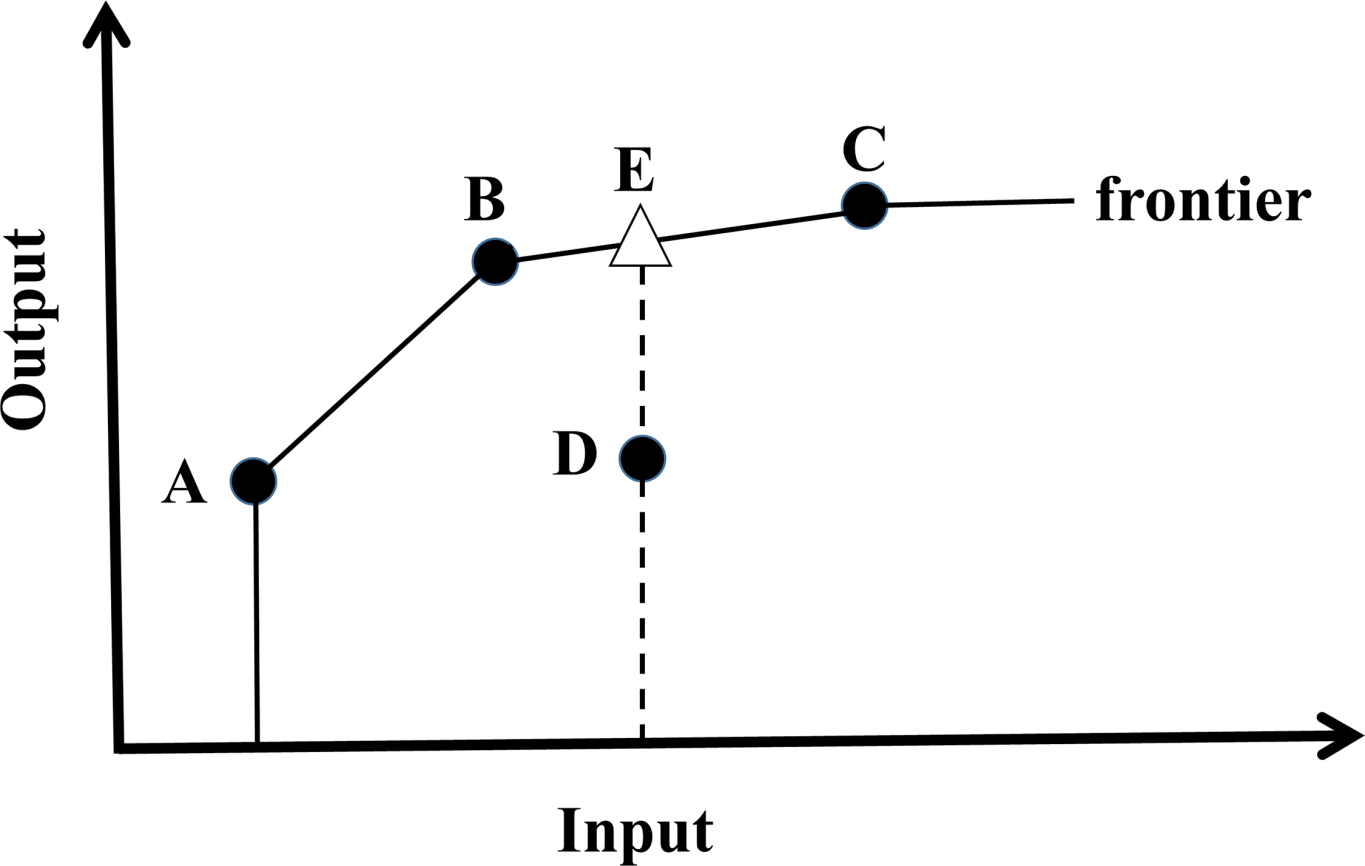
Diagram of best-practice frontier defined assuming variable returns to scale (modified from FAO 2003 [6]).

Fig 1 illustrates the construction of the best-practice frontier for a single input–single output case assuming variable returns to scale (VRS). Variable returns to scale indicates that as the input is changed, the output can be increasing, decreasing, or constant with the proportional change in input. Under the VRS assumption, the best-practice frontier is defined by points A, B, and C, with these three points having an efficiency of 100%. Point D lies below the frontier and is considered to be inefficient (<100% efficiency), with point E corresponding to the output that would result in 100% efficiency for the given input of D.

Data envelopment analysis has been used by Fukuda-Parr to construct a Social and Economic Rights Fulfillment (SERF) index [7], which identifies the possible achievements of a country given their national income (measured as GDP per capita). However, the SERF index focuses on a country’s status (e.g., percent of the population with access to improved water) and does not consider rates of change. In this paper, we use a new application of frontier analysis, previously developed and demonstrated with water and sanitation [3], to identify the best-practice (i.e., benchmark) rates of change given a country’s status and we use these benchmark rates to assess country performance through a performance index.

## Constructing a Performance Index Using Frontier Analysis

We construct performance indices for three MDG and SDG targets. The targets were selected based on data availability and to reflect public health (e.g., maternal mortality), human rights (e.g., education), and international development (e.g., poverty). We note that performance indices can be calculated, where data is available, for other health targets such as cessation in smoking or specific vaccine immunizations, as well as for indicators related to the MDGs or SDGs. Using data from the World Bank’s World Development Indicators [2], we calculate performance indices for indicators 1–3 listed in Table 1. In addition to the indicator, the corresponding MDG and SDG are also provided for reference. Table 1 also includes a fourth indicator on previously reported progress towards equity in rural-to-urban water access which uses frontier analysis [3]. The data sources used to calculate performance indices are estimates taken from nationally representative household surveys such as Demographic and Health Surveys (DHS) and national censuses. If possible, modeled estimates should be avoided as these values have already undergone some form of regression. For example, to calculate the performance index for indicator 4, original national survey data [8] was used instead of the estimates provided by the WHO/UNICEF Joint Monitoring Programme.

**Table 1:** Indicators for which performance indices are calculated.

#	Indicator	Corresponding MDG Target^a^	Corresponding SDG Target^b^
1	Poverty headcount ratio at $1.25 a day (PPP) (% of population)^c^	Target 1A: halve, between 1990 and 2015, the proportion of people whose income is less than $1.25 a day	Target 1.1: by 2030, eradicate extreme poverty for all people everywhere, currently measured as people living on less than $1.25 a day
2	Primary school completion rate, total (% of relevant age group)^d^	Target 2A: ensure that, by 2015, children everywhere, boys and girls alike, will be able to complete a full course of primary schooling	Target 4.1: by 2030, ensure that all girls and boys complete free, equitable and quality primary and secondary education leading to relevant and effective learning outcomes
3	Maternal mortality ratio (national estimate, per 100,000 live births)^e^	Target 5A: reduce by three quarters, between 1990 and 2015, the maternal mortality ratio	Target 3.1: by 2030, reduce the global maternal mortality ratio to less than 70 per 100,000 live births
4	Equitable access to safe drinking water between rural and urban areas^f^	Target 7C: halve, by 2015, the proportion of the population without sustainable access to safe drinking water and basic sanitation	Target 6.1: by 2030, achieve universal and equitable access to safe and affordable drinking water for all

^a^ Paraphrased from the United Nations [1,9].

^b^ Paraphrased from the United Nations [10].

^c^ $1.25 at 2005 international prices. Data are from primary household survey data.

^d^ Measured as the total number of students in the last grade of primary school, minus the number of repeaters in that grade, divided the total number of students of official graduation age. For values that were >100%, these were manually changed to 100%. Data are obtained from national statistical offices submitted to the UNESCO Institute for Statistics (UIS), with the UIS sometimes generating estimates and imputing missing data

^e^ Measured as the number of women who die from pregnancy-related causes while pregnant or within 42 days or pregnancy termination per 100,000 live births. These are national estimates.

^f^ Definitions of ‘safe’ and ‘basic’ as determined by the use of ‘improved’ technologies defined by the WHO/UNICEF Joint Monitoring Programme (JMP)

We use the indicator “primary school completion rate” as our example calculation and divide this section into the following: defining the metric of interest and its corresponding target, calculating rates of change, using DEA to identify frontier points and constructing the best-practice frontier, and calculating performance indices and interpreting the results. Our analysis is performed in R [11] using the FEAR [12] software package for DEA and outlier identification, although STATA, SAS, and specialized software packages are also available.

### Defining the metric of interest and its corresponding target

In the case of primary school completion rate, we are interested in whether a country is making progress towards achieving universal coverage for primary school completion, and as such, our metric of interest is the percent of primary-age school children who have completed all levels of primary school. However, if our interest were whether there is equal achievement of primary school completion between boys and girls, our metric of interest would be the ratio of completion between girls-to-boys. It is important to properly identify the metric of interest as this is used as the *x*-axis parameter when constructing the DEA figures. In addition to defining the metric of interest, the corresponding target should also be defined. When considering progress towards achieving *universal access* to, for example, primary school completion or improved drinking water and sanitation, the target is 100% of the population. However, for indicators such as maternal mortality ratio, where a *lower ratio* of maternal deaths to live births is desired, the target would be zero. For metrics that focus on equity and consist of a *ratio between two percentages* (e.g., girls-to-boys primary school completion rate, poorest-to-richest wealth quintile’s access to basic sanitation), the corresponding target would be 1 to reflect equal access/achievement. Defining the target value for each metric of interest is needed in order to determine whether the calculated rates of change correspond to positive performance (i.e., progression) or negative performance (i.e., regression).

### Calculating rates of change

To assess progress, linear rates of change are calculated to describe how the metric of interest changes with time. The criteria for data inclusion depends on the overall objective of the study as well as data availability. For example, if the objective of a study is to identify the underlying drivers of progress towards increasing access to safe water and basic sanitation since the MDGs were set in 2000, all data points from 2000–2012 would be used to calculate the rates of change. In this paper, we use a set of general criteria that rates of change must be calculated with a minimum of five data points (to reduce the variability in survey data) that span at least three different years. Countries that do not meet this criteria were not included in the analysis. Additional criteria that can be imposed include the range of years that the data points span (e.g., data points should not exceed a 10-year period), visual inspection of each calculated rate to ensure that a linear fit is appropriate, and a decision on whether data from the same year is counted as separate data points or averaged. Countries that have undergone internal or external conflict can also be identified and excluded from all analyses or only excluded from defining the frontier, as was the case by Fukuda-Parr *et al* [7].

Once rates of change are calculated, these rates need to be associated to a corresponding metric of interest. For example, Burundi’s primary school completion rates for the years 2008–2012 were 41.3%, 47.8%, 50.8%, 55.5%, and 62.2%. The rate of change is calculated by fitting a straight line through these points and obtaining the slope, which is +4.9%/year. The corresponding year and primary school completion rate is selected to be the average year of 2010 and the average primary school completion rate of 51.5%. Note that if our interest were to compare rates of change between countries with the same *initial* primary school completion rate, we would select our corresponding year and primary school completion rate to be 2008 and 41.3%, respectively.

### Using data envelopment analysis to identify frontier points and constructing the best-practice frontier

**Fig 2:**
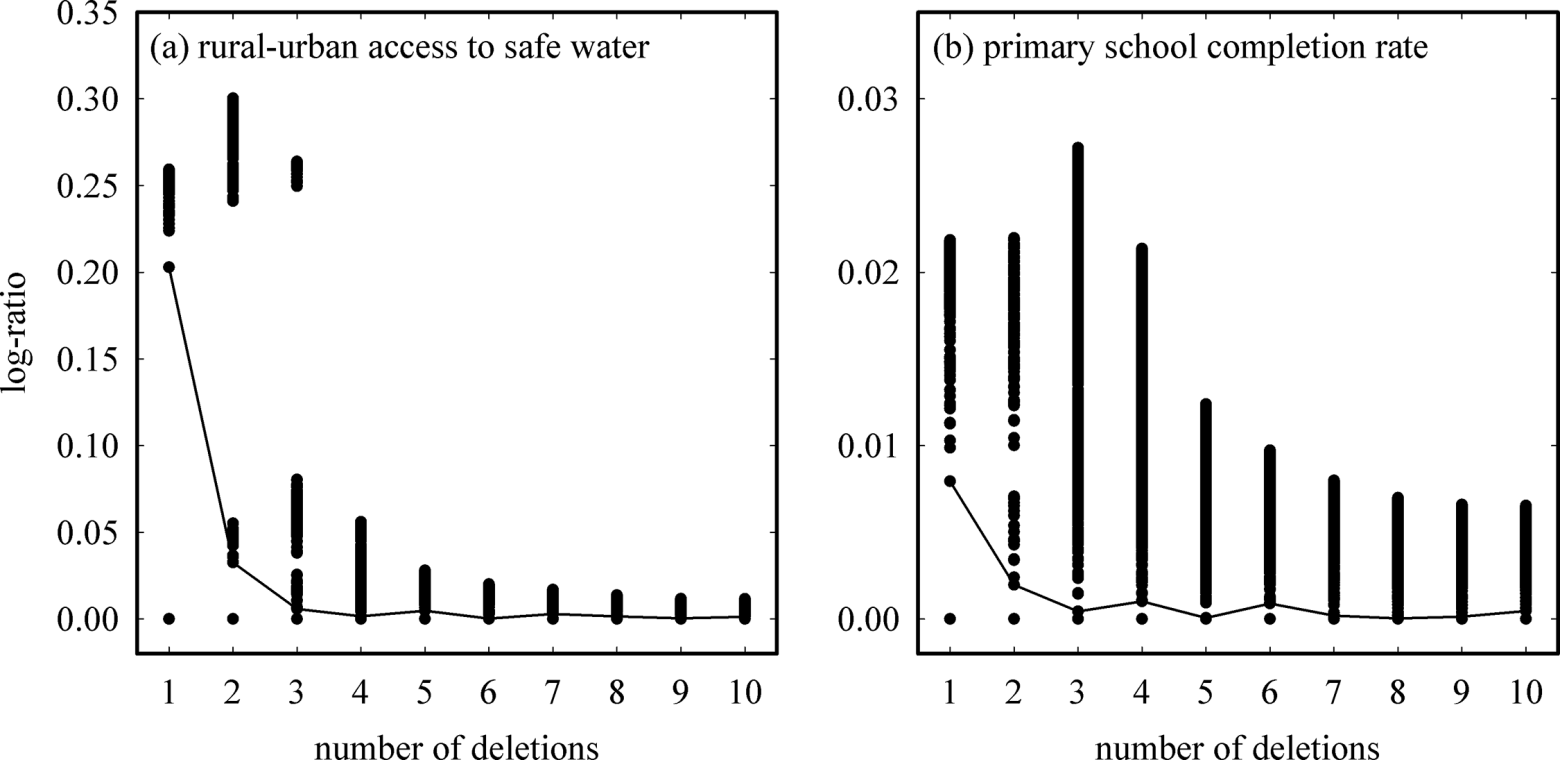
Outlier detection plots using the “ap.plot” command from FEAR in R for (a) rural-to-urban water access [3] and (b) primary school completion rate. Each point corresponds to the reduction in geometric space resulting from a given number of deletions. The line identifies the greatest reduction at each deletion value.

Prior to identifying the frontier points and constructing the best-practice frontier, the “ap” and “ap.plot” commands in R’s FEAR software package are used to identify outliers. Outlier identification and removal is critical because they can greatly influence the best-practice frontier and thus the calculated performance of each country. We used Wilson’s method [13], based on the work of Andrews and Pregibon [14], as the outlier detection method in FEAR. Briefly, the geometric space that all points occupy is first calculated. The software then calculates the geometric space occupied for all possible combinations of data points with one deletion, two deletions, and up to a user-specified number of deletions. The “ap.plot” command generates a plot describing these results where the reduction in geometric space resulting from the deletions is given by the log-ratio value, and *% reduction* = (1 – 10^*log-ratio*^)×100%. The number of outliers detected corresponds to the number of deletions that results in the greatest reduction in geometric space. For convenience, the software draws a line in the plot to identify the greatest reduction at each deletion value and the user looks for the deletion value with the largest log-ratio. It should be noted that running the “ap” and “ap.plot” commands will always return outlier detection results and thus the user must specify the cutoff in terms of reduction in geometric space that determines whether a true outlier is identified. For example, Fig 2 shows sample plots for outlier detection for the case of (a) rural-urban access to water [3] and (b) primary school completion rate. As seen in Fig 2A, the largest log-ratio gap of 0.20 and therefore greatest reduction in geometric space (at 58%) occurred in the case of one deletion. While Fig 2B also identified one deletion as corresponding to the greatest reduction in geometric space, the associated log-ratio was only 0.08 (and a 20% reduction). We chose to use a log-ratio of 0.1 (corresponding to a 26% reduction) as the cutoff and therefore no outliers were identified for primary school completion rate from Fig 2B.

**Fig 3:**
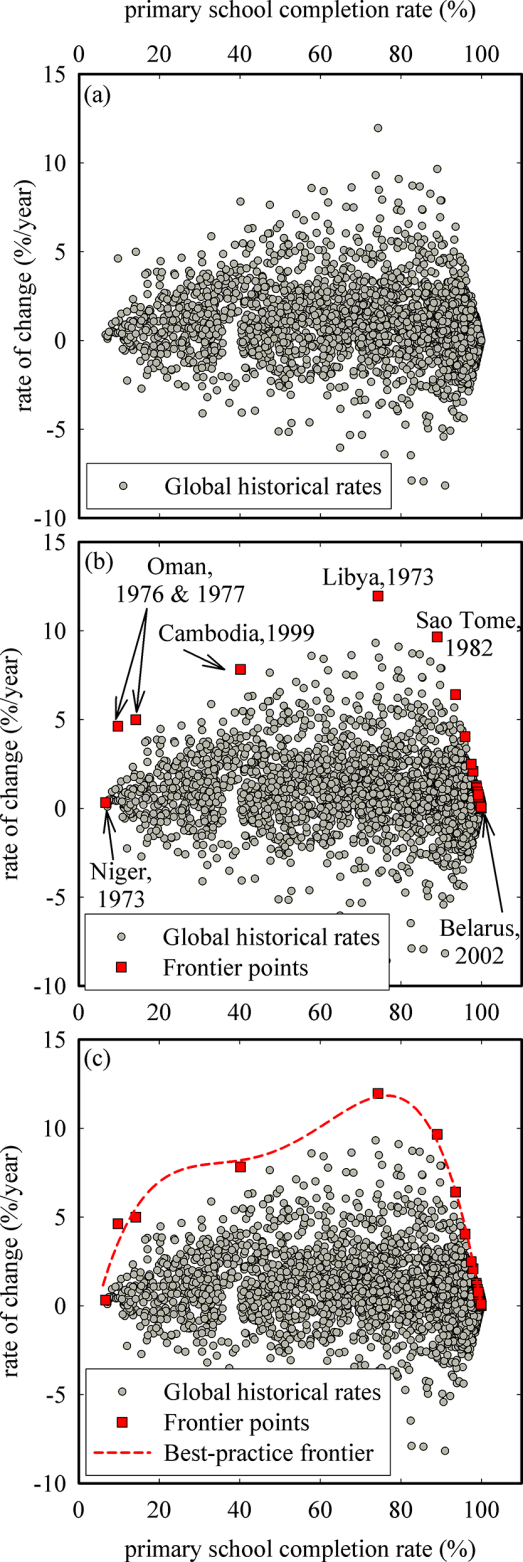
Identification of frontier points and construction of the best-practice frontier for primary school completion rate.

Following the detection and subsequent removal of outliers, the “dea” command in R is used to identify the frontier points. Fig 3A shows all calculated historical rates of change plotted as a function of primary school completion rate. The frontier points identified by the “dea” command are highlighted as red squares in Fig 3B and are predominantly found on the downward part of the curve. Since data envelopment analysis is used for the identification of frontier points, only the points that form the ‘envelope’ are considered to be frontier points. As such, there can be large gaps between two consecutive frontier points, for example from Oman 1977 to Cambodia 1999 and from Cambodia 1999 to Libya 1973, because the data points in between the two consecutive frontier points fall below the ‘envelope’. A polynomial equation is then fit to the frontier points to obtain the best-practice frontier (red dotted line in Fig 3C), with the constraint that the rate of change must be equal to zero for primary school completion rates of 100%. Similarly, for indicators where a zero target is desired, the rate of change should be zero once the zero target (e.g., maternal mortality rate) is reached. The construction of the best-practice frontier now allows “virtual” frontier points to be calculated at every point along the *x*-axis.

### Calculating performance indices and interpreting the results

The performance a country makes towards achieving universal coverage for primary school completion is calculated using a modified version of the formula used to calculate efficiency in standard DEA. Typically, in DEA, outputs are greater than zero and therefore there is no need to consider negative outputs (i.e., regression). To reflect the positive and negative potential nature of a country’s rate of change, we followed the United Nations Development Programme [15] towards calculating indices and define both a minimum and maximum rate of change as shown below:

$$index=\frac{country\;rate-minimum\;rate}{maximum\;rate-minimum\;rate},$$ (1)

The maximum rate is defined as the maximum possible rate achievable, or benchmark rate, at the coverage level of the country analyzed and is calculated from the best-practice frontier, and the minimum rate is defined as zero (no progress). For countries with negative rates of change, use of Eq 1 will result in a negative performance index.

**Fig 4:**
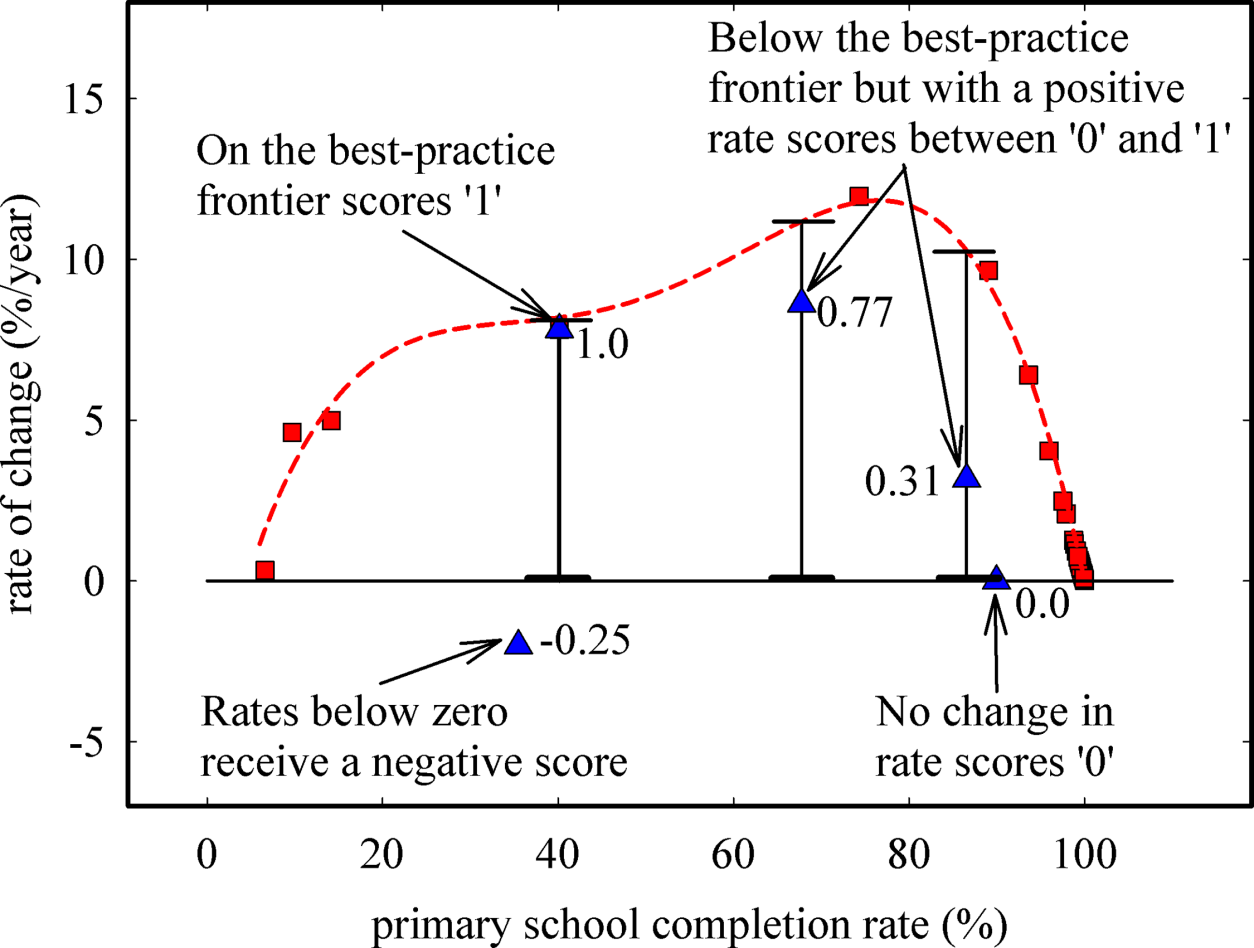
Calculation of performance indices for Cambodia at five points in time. Frontier points are shown as squares, the best-practice frontier is given by the dotted line, and the line of no progress is given by the solid horizontal line. Triangles correspond to five example rates of change for Cambodia, with the associated performance index given as numerical values next to the triangles.

Fig 4 illustrates how the performance index of Cambodia changes with time using five example rates of change, which allows a country to perform self-assessments of progress over time. The frontier points (red squares), best-practice frontier (dotted red line), and no-progress line corresponding to a rate of 0%/year (black horizontal line) are shown for reference. Four regions can be defined in Fig 4: points that fall on the best-practice frontier are the best-performers and have performance index scores of 1; points that fall below the best-practice frontier but above the no-progress line have performance indices between 0 and 1; points on the no-progress line, which can be defined as rates of change between -0.05%/year and +0.05%/year (or -0.1 to +0.1%/year depending on the user) score a value of 0; and points below the no-progress line will have negative performance index scores. As shown in Fig 4, performance index scores of -0.25, 1.0, 0.77, 0.31, and 0.0 were calculated for the average years of 1996, 1999, 2003, 2006, and 2008, respectively, indicating that during the late-1990s to mid-2000s, Cambodia made progress towards universal coverage for primary school completion; however the performance index of 0.0 in recent years shows that no progress was made in that time period.

By using DEA to construct a best-practice frontier, benchmark (maximum achievable) rates at all values of primary school completion levels can be determined and used to calculate performance indices. Table 2 gives the performance indices for primary school completion for the year 2009, along with the corresponding status (i.e., level of attainment or coverage) and trend in status (i.e., rate of change), for all countries with available data. These countries can now be fairly compared against each other without needing to specify their primary school completion level for 2009. For example, from Table 2, we see that both Costa Rica and Djibouti have performance index values of 0.31, indicating that they are making the same progress towards achieving universal coverage for primary school completion despite their different primary school completion levels (Costa Rica at 95.2% and Djibouti at 43.8%). The index can also be used to identify countries at similar rates of change and compare their performance. For example, while Guatemala and Djibouti both have rates of change of ~2.6%/year (with primary school completion levels of 83.0 and 43.8%, respectively), Djibouti has been making greater progress as seen by its performance index of 0.31, as compared to Guatemala with an index of 0.23.

**Table 2:** Performance indices calculated for the year 2009 for indicators 1–3. Results of indicator 4 (for the year 2010) are taken from Luh *et al*. [3]. For countries where indices were not available for 2009, we provide the values for the closet year, with the year in brackets.

Country	Proportion of the population whose income is less than $1.25 a day (2009)			Primary school completion rate (2009)			Maternal mortality ratio (2009)			Equity in rural-urban access to safe drinking water (2010)		
	Avg Status ^a^ (%)	Trend in Status ^b^ (%/yr)	Index	Avg Status ^a^ (%)	Trend in Status (%/yr)	Index	Avg Status ^a^	Trend in Status ^b^	Index	Avg Status ^a^	Trend in Status	Index
Algeria				90.8	1.65	0.20						
Argentina	2.31	-0.42	0.33	100	0	n/a	44.6	-0.40	0.06			
Armenia	2.26	-0.08	0.06				16.2	-0.36	0.09 (2008)	0.93	0.02	0.64
Aruba				95.0	0.39	0.07 (2008)						
Austria				98.9	-0.70	-0.53						
Azerbaijan				92.6	-1.19	-0.17						
Bahamas				98.6	-1.37	-0.82 (2008)						
Bangladesh				65.3	1.57	0.15						
Barbados				97.5	1.79	0.61						
Belarus	0.00	0	0	98.7	1.13	0.70	2.4	-0.91	0.65			
Belgium				88.6	1.41	0.15						
Belize				100	0	n/a				0.89	0.02	0.47
Benin				63.5	2.48	0.23				0.77	0.00	0.02
Bhutan				84.7	4.00	0.37						
Bolivia	9.66	-0.95	0.22	94.1	-1.18	-0.19				0.66	0.03	0.54
Brazil	4.74	-0.33	0.14							0.84	0.01	0.31
Brunei Darussalam				100	0	n/a						
Bulgaria	1.03	0.17	-0.30 (2008)	96.3	1.52	0.37	5.78	-0.51	0.19			
Burkina Faso				44.5	4.50	0.54				0.74	-0.01	-0.23
Burundi				46.8	4.36	0.51						
Cabo Verde				96.7	1.04	0.28				0.95	-0.00	-0.03
Cambodia	17.2	-5.12	0.84	90.8	0.44	0.05				0.67	-0.01	-0.26
Cameroon				65.4	3.46	0.32						
Central African Republic				38.6	2.40	0.29						
Chad				32.5	0.92	0.12 (2010)				0.69	-0.01	-0.29
Chile				96.2	0.28	0.07	18.6	-0.18	0.04 (2008)			
China							31.8	-2.52	0.47	0.73	0.02	0.47
Colombia	7.14	-1.01	0.30	100	0	n/a				0.70	0.00	0.10
Congo, Dem. Rep.				61.7	2.74	0.27						
Congo, Rep.				72.3	-0.08	-0.01						
Costa Rica	1.97	-0.24	0.22	95.2	1.59	0.31	28.1	-2.60	0.52	0.91	0.00	0.15
Cote d’Ivoire				53.5	2.32	0.25				0.76	-0.01	-0.17
Croatia				95.1	-1.84	-0.35	10.8	-0.63	0.19			
Cuba				94.8	2.33	0.42	36.3	5.22	-0.91			
Cyprus				99.9	-0.06	-0.31						
Czech Republic	0.04	-0.01	0.30	99.2	0.77	0.80						
Denmark				99.4	-0.15	-0.18						
Djibouti				43.8	2.59	0.31						
Dominica				88.7	-0.97	-0.10						
Dominican Republic	2.97	-0.35	0.22	89.3	1.26	0.14						
Ecuador	5.57	-0.75	0.27	100	0	n/a						
Egypt				99.3	0.45	0.49				0.98	0.00	0.23
El Salvador	3.98	0.03	-0.01	93.2	2.84	0.42	81.4	-11.0	1.0 (2008)			
Equatorial Guinea				49.3	0.96	0.11						
Eritrea				38.9	-2.43	-0.30						
Estonia	0.66	0.14	-0.37	96.7	-0.87	-0.23						
Fiji				100	0	n/a						
Finland				97.9	-0.21	-0.08						
Gambia				76.5	-2.35	-0.20						
Georgia	15.4	0.66	-0.11	98.9	0.79	0.60	27.2	1.26	-0.26			
Germany				100	0	n/a						
Ghana				87.6	3.87	0.39				0.73	0.02	0.43
Greece				99.6	-0.21	-0.47 (2008)						
Grenada				99.5	0.13	0.21 (2008)						
Guatemala				83.0	2.61	0.23						
Guinea				58.0	0.92	0.09						
Guinea-Bissau										0.63	-0.01	-0.18
Guyana				95.5	-2.20	-0.45				0.94	0.00	0.07
Honduras	14.5	0.02	-0.00	94.0	2.43	0.40						
Hong Kong				97.1	-0.79	-0.24						
Hungary	0.05	-0.00	0.01 (2008)	97.8	0.54	0.21						
Iceland				97.7	0.36	0.15						
India										0.90	0.00	0.13
Indonesia				99.4	0.39	0.48				0.79	0.01	0.13
Iran				98.8	1.02	0.70						
Israel				100	0	n/a						
Italy				100	0	n/a						
Japan				100	0	n/a						
Jordan										0.93	0.01	0.20
Kazakhstan				100	0.04	0.78						
Kenya										0.56	0.02	0.31
Korea, Rep.				99.9	0.05	0.39						
Kyrgyz Republic	4.83	0.69	-0.28	96.3	-0.05	-0.01						
Laos				80.1	4.05	0.35				0.63	0.03	0.63
Latvia	0.75	0.12	-0.28 (2008)	94.1	1.70	0.28						
Lebanon				84.5	0.72	0.07						
Lesotho				72.8	-0.36	-0.03				0.81	-0.00	-0.03
Liechtenstein				98.9	0	0						
Lithuania	1.09	-0.10	0.16 (2008)	99.4	0.29	0.40						
Macedonia				89.0	-2.87	-0.31 (2008)						
Madagascar				68.7	2.34	0.21				0.41	0.02	0.39
Malawi				65.1	2.80	0.26				0.79	0.02	0.53
Malaysia							28.8	-0.68	0.13 (2008)			
Mali				58.9	2.01	0.20						
Malta				92.0	-0.90	-0.12						
Marshall Islands				100	-0.05	-0.74 (2008)						
Mauritania				55.4	4.15	0.44 (2008)						
Mauritius				99.7	0	0						
Mexico	1.70	-0.37	0.40 (2008)	94.0	-1.01	-0.16	55.1	-2.84	0.39	0.90	0.01	0.30
Moldova	0.70	-0.22	0.56	92.0	-0.30	-0.04	29.0	0.57	-0.11 (2010)	0.94	0.00	0.12
Mongolia				100	0	n/a	72.0	-8.19	1.0 (2008)			
Montenegro	0.13	-0.04	0.53									
Morocco				83.0	1.77	0.16						
Mozambique				55.3	2.18	0.23				0.37	0.01	0.14
Myanmar				91.1	1.66	0.20 (2008)				0.71	0.01	0.29
Namibia				82.4	0.58	0.05						
Nepal				88.7	2.76	0.29				0.89	-0.00	-0.10
Niger				41.3	1.22	0.15				0.45	-0.01	-0.29
Nigeria				77.9	-3.63	-0.31 (2008)				0.52	0.03	0.54
Norway				98.9	-0.07	-0.05						
Oman							19.1	0.76	-0.18 (2008)			
Pakistan				63.4	1.84	0.17				0.91	0.02	0.57
Panama	4.46	-0.82	0.36	94.7	0.15	0.03						
Paraguay	5.45	-0.51	0.19	91.8	-2.05	-0.27						
Peru	4.07	-0.88	0.42	98.6	-0.87	-0.52				0.67	0.00	0.02
Poland	0.01	-0.01	1.0	95.3	-0.02	-0.00						
Romania	0	0	n/a	95.1	-0.42	-0.08						
Russian Federation				95.0	1.16	0.22						
Rwanda				50.1	1.89	0.21				0.76	0.01	0.26
Samoa				100	0	n/a						
Sao Tome and Principe				85.3	6.45	0.60						
Saudi Arabia				94.6	1.87	0.33						
Senegal				57.3	2.10	0.22				0.57	0.00	0.06
Serbia	0.11	-0.05	0.74 (2008)	98.4	-0.49	-0.26						
Seychelles				100	0	n/a						
Sierra Leone										0.40	-0.00	-0.02
Slovak Republic	0.18	0.13	-1.3	98.5	0.58	0.32						
Slovenia	0.03	-0.01	0.71 (2008)	96.6	0.94	0.24 (2010)						
South Africa										0.76	0.02	0.34
Spain				99.7	0.26	0.79						
Sri Lanka				95.8	1.47	0.32						
St. Kitts and Nevis				90.6	0.38	0.05						
St. Lucia				95.2	-2.32	-0.45						
St. Vincent and the Grenadines				96.1	-0.97	-0.23						
Sudan										0.72	0.00	0.08
Suriname				85.7	0.25	0.02 (2008)						
Swaziland				73.1	1.80	0.15						
Sweden				95.8	1.23	0.27						
Switzerland				95.1	0.84	0.16						
Syrian Arab Republic				100	0	n/a						
Tajikistan				96.9	2.06	0.59						
Tanzania				85.1	1.48	0.14				0.54	0.00	0.06
Timor-Leste										0.69	-0.00	-0.08
Togo				70.2	0.11	0.01				0.45	-0.00	-0.01
Trinidad and Tobago				94.3	0.55	0.09 (2008)						
Tunisia				96.4	-0.87	-0.22 (2008)						
Turkey	0.42	-0.13	0.53	99.5	0.45	0.70				0.88	0.01	0.31
Uganda				54.7	0.09	0.01				0.66	0.02	0.32
Ukraine	0.05	-0.03	0.94 (2008)	98.7	0.02	0.02						
United Arab Emirates				96.1	1.66	0.38						
United States				98.0	-0.16	-0.06						
Uruguay	0.29	-0.06	0.31	99.9	0.08	0.78 (2008)						
Uzbekistan				93.7	-1.44	-0.23						
Vanuatu				84.7	-1.21	-0.11 (2008)						
Venezuela				95.2	-0.68	-0.13						
Vietnam	15.2	-3.77	0.66 (2008)							0.85	0.01	0.32
West Bank and Gaza				89.7	1.51	0.17						
Yemen				63.8	1.09	0.10 (2008)						
Zambia				93.5	0.07	0.01						
Zimbabwe										0.71	-0.01	-0.14

^a^ Avg Status = taken as the average of the 5 data points

^b^ For Trend in Status for Proportion of the population whose income is less than $1.25 a day and Maternal mortality ratio, a negative trend is the desired outcome. For example, for maternal mortality ratio, a negative trend indicates that there is a decrease in the number of women who die from pregnancy-related causes while pregnant or within 42 days or pregnancy termination per 100,000 live births

**Fig 5:**
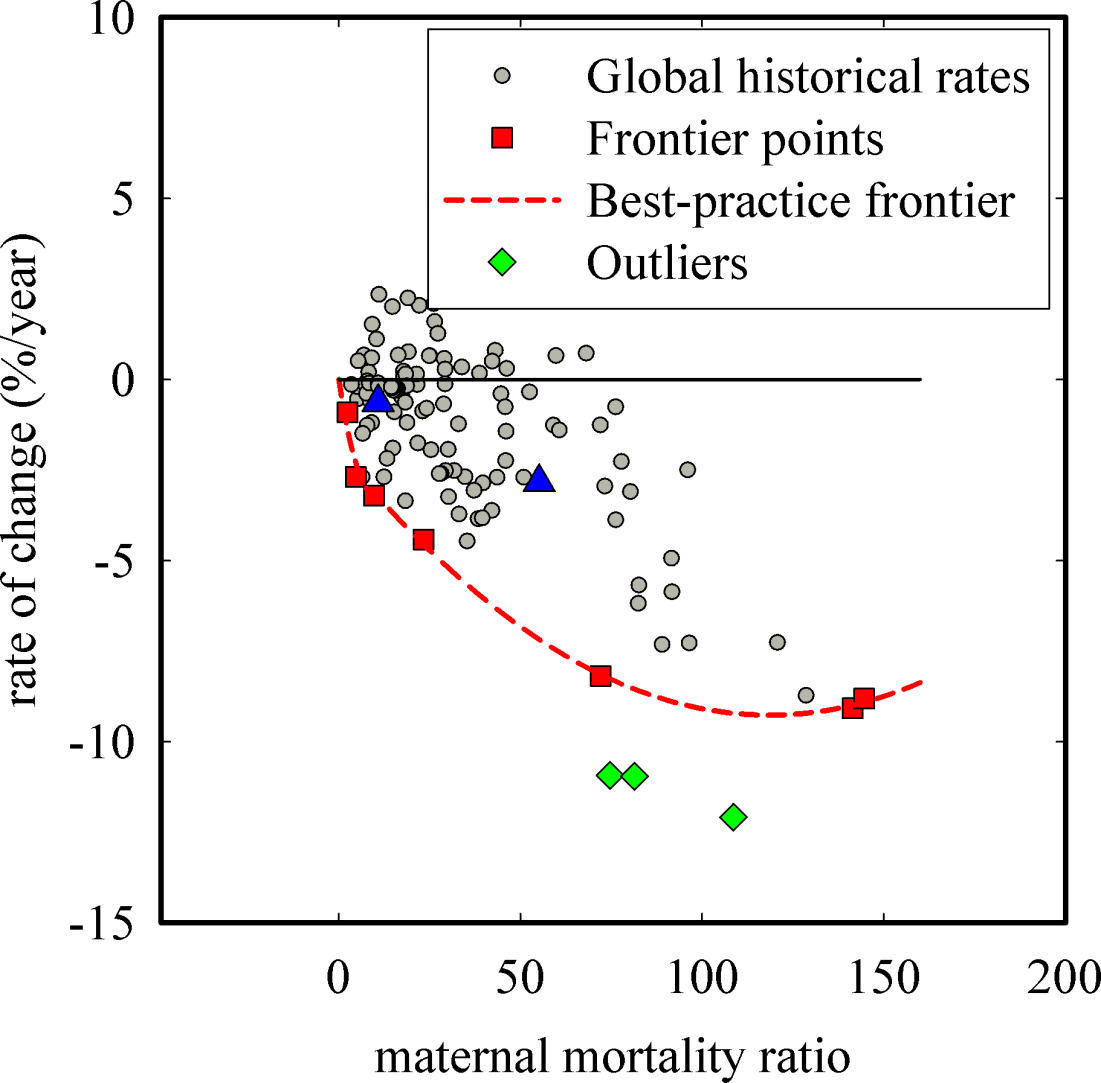
Construction of the best-practice frontier and calculation of performance indices for maternal mortality ratio. Global historical rates calculated from available data are shown as circles, frontier points are shown as squares, the best-practice frontier is given by the dotted line, and the line of no progress is given by the solid horizontal line. Three outliers (diamonds) were identified through FEAR. Points A and B (triangles) correspond to two example rates of change with the associated performance index given as numerical values next to the triangles.

For indicators where a zero target is desired (e.g., maternal mortality ratio where a lower number of maternal deaths to live births is the objective), a negative rate of change indicates that progress is being made (i.e., a decrease in status is observed). Fig 5 illustrates the global historical rates (grey circles), frontier points (red squares), best-practice frontier (dotted red line), outliers as identified by FEAR (green diamonds), and no-progress line corresponding to a rate of 0%/year (black horizontal line) for the indicator of maternal mortality ratio. Seven frontier points were identified by the “dea” command, corresponding to Belarus 2009, 2008, and 2006, Moldova 2004, Mongolia 2008, Guyana 2004, and Paraguay 2006. The best-practice frontier provides the maximum achievable rates, which are negative rates, and Eq 1 is used to calculate the performance indices, with performance index values for 2009 given in Table 2. Points A and B in Fig 5, which correspond to Croatia 2009 and Mexico 2009, respectively, demonstrate how country comparisons can be made. Despite the higher maternal mortality ratio observed in Mexico (55.1 as compared to 10.8 of Croatia), Mexico has a performance index of 0.39 and therefore has made greater progress than Croatia (with a performance index of 0.19) to lower its maternal mortality.

## Discussion

This paper describes the application of frontier analysis to calculate performance indices which can be used to assess and compare progress made by countries towards public health, human rights, and international development goals. The method uses the principles of data envelopment analysis to identify the historically best-performing countries, which are then used to define benchmark rates of progress. The main advantage of this method over established measures of progress that focus on status (level of attainment or coverage) or rates of change is that the level of development of a country is taken into account by considering both status and rates of change. Compared with other indicators of progress, performance indices calculated using frontier analysis allow countries to be fairly compared against each other regardless of whether they have only just begun to make progress or whether they have almost achieved the target. The performance index thus complements existing indicators of progress. As a specific example, we see from Table 2 that in 2009, using status alone, Benin would be considered to be making greater progress than Central African Republic (CAR), with primary school completion levels of 63.5% and 38.6%, respectively. Furthermore, the rates of change in primary school completion also support Benin as the country making greater progress, at 2.48 and 2.40%/year for Benin and CAR, respectively. However, our performance index, which recognizes the fact that rates of change are dependent on status, shows that the progress CAR has achieved is, in fact, greater than that achieved by Benin.

The computations required to calculate a performance index are more complex than established indicators of progress; however, these indices have the advantage that they can be used to make comparisons across settings (e.g., international–country-to-country, sub-national–regional or county level, facilities–water supply and health care) and over time (e.g., monitoring progress of a single country over time). In addition to comparisons, performance indices calculated using frontier analysis also identify the unfulfilled potential a country has to most effectively use its resources to achieve the greatest possible progress. Therefore, the concept used to assess progress aligns with the principle of progressive realization for all human rights. This principle recognizes that countries are limited by prior conditions and resources available and therefore compliance with the human right is achieved as long as countries show they are using the maximum resources available to take steps towards the human right.

We demonstrate the calculation of performance indices for three indicators in this paper, all three with targets of either universal access or zero deprivation. However, this method can also be applied to assess equity amongst different population groups. For example, the results of rural-urban disparity in access to safe drinking water from Luh *et al*. [3] are summarized as indicator 4 in Table 2 and the index values were previously shown to have no correlation with factors such as gross national income per capita. Additional population groups can be compared, such as different wealth quintiles or ethnic groups depending on data availability. Fields in which this method can be applied are not limited to those identified by the MDGs or SDGs, but can also focus on specific health targets such as cessation in smoking or specific vaccine immunizations.
